# Anterior Cruciate Ligament Reconstruction and Concomitant Focal Cartilage Lesions: A Systematic Review and Meta-Analysis of Prognosis after Surgical Treatment

**DOI:** 10.1177/19476035241292719

**Published:** 2024-11-20

**Authors:** Diko Jevremovic, Asbjørn Årøen, Owen Matthew Truscott Thomas, Hilde Moseby Berge, Ahsan Ayub Khan, Svend Ulstein

**Affiliations:** 1 Institute for Health and Society, Medical Faculty, University of Oslo, Oslo, Norway; 2 Department of Orthopedic Surgery, Kristiansund Hospital, Kristiansund, Norway; 3 Institute of Clinical Medicine, University of Oslo, Oslo, Norway; 4 Oslo Sports Trauma Research Centre, Norwegian School of Sport Sciences, Oslo, Norway; 5 Department of Orthopedic Surgery, Akershus University Hospital, Lørenskog, Norway; 6 Department of Health Services Research, Akershus University Hospital, Lørenskog, Norway; 7 Department of Sports Medicine, Oslo Sports Trauma Research Center, Norwegian School of Sport Sciences, Oslo, Norway; 8 Department of Sports Medicine, Norwegian Olympic Sports Center Olympiatoppen, Oslo, Norway

**Keywords:** anterior cruciate ligament, ACL, cartilage, lesions, PROMS

## Abstract

**Objective:**

To synthesize available evidence on the impact of concomitant focal cartilage lesions and their surgical treatment on clinical outcomes in the setting of anterior cruciate ligament (ACL)-reconstruction at short (6-36 months) and midterm (3-8 years) follow-up.

**Design:**

Original level 1 or 2 studies comparing any patient-reported or objective outcomes in ACL-reconstructed patients (1) with and without concomitant focal cartilage lesion(s) or (2) after any type of cartilage surgical treatment were considered for inclusion. Systematic searches were conducted in MEDLINE via Ovid, Cochrane Library, EMBASE via OvidSP, and Web of Science.

**Results:**

In meta-analysis performed across 6 studies (n=8,789 patients), we discovered with very low certainty, the correlation of concomitant any-thickness cartilage lesions and worse Patient-Reported Outcome Measure scores (PROMS) at cumulative short to mid, (pooled standardized mean difference (psmd) = −0.36; 95% confidence interval (CI) –0.62 to −0.10), short (psmd = −0.43; 95% CI = −0.94 to 0.08), and midterm (psmd = −0.22; 95% CI –0.43 to 0.00). Full-thickness lesions predicted worse PROMS with moderate certainty at cumulative short-midterm (psmd = −0.32; 95% CI = −0.41 to −0.23) and low certainty at both short (psmd = −0.45; 95% CI –0.83 to −0.07) and midterm (psmd = −0.30; 95% CI –0.38 to −0.22). In 4 studies for each outcome, mixed results were reported on osteoarthritis (OA) and reoperation rates.

**Conclusions:**

As the main finding, concomitant full-thickness cartilage lesions in ACL-reconstructed patients are a predictor of worse PROMS in the cumulative short to midterm. Correlations of any-thickness lesions or different cartilage treatments with short- or midterm PROMS, OA, or reoperation rates were either with very low certainty, unmeasured, or with mixed results.

## Introduction

The anterior cruciate ligament (ACL) is a functionally important component of a healthy knee joint. Due to high functional demands, acute tears of the ACL are common, especially in young, athletic individuals. The reported incidence ranges up to 68.8 cases per 100,000 person-years worldwide.^1-4^ ACL rupture is characterized in the short term by pain, instability, and reduced range of motion due to effusion^5,6^ and in the long term by potential premature emergence and accelerated progression of osteoarthritic changes.^7-9^ Concomitant focal cartilage lesions are an important predictor of functional outcomes after ACL reconstruction,^10,11^ detected in 19%-36% of all patients undergoing ACL reconstruction.^12-14^ Although cartilage lesions contribute to knee osteoarthritis (OA) development in the long term,^15,16^ their influence on Patient-Reported Outcome Measure Scores (PROMs) is still not well understood.^17-20^

Between 2017 and 2021, 4 systematic reviews (SRs) were published^21-24^ on PROMs or objective outcomes at short- to midterm follow-up after ACL reconstruction in patients with cartilage lesion(s). The majority of the existing SRs on this topic did not restrict themselves to studies with higher levels of evidence, or the Grading of Recommendation, Assessment, Development, and Evaluation (GRADE) Working Group’s guidelines for grading the quality of evidence.^21,22^ Notably, none of the relevant published SRs performed quantitative synthesis of results in a meta-analysis. Consequently, neither the prognostic value nor the optimal treatment of cartilage lesions in ACL-reconstructed patients is well established.

Identification of the short- and midterm clinical outcomes after ACL reconstruction in patients with and without cartilage lesions was determined as the primary study objective. Identification of the preferred surgical approach for treating cartilage lesions at the time of ACL reconstruction was determined as a secondary study objective.

## Methods

This SR and meta-analysis was performed following a predefined protocol complying with recommendations from the Cochrane Musculoskeletal Group,^25^ and designed following the Preferred Reporting Items for Systematic Reviews and Meta-analysis (PRISMA-P) guidelines.^26^

### Inclusion and Exclusion Criteria

Original level 1 or 2 studies^27^ reporting outcomes after ACL reconstruction in patients with cartilage lesions, comparing either different surgical treatments, or to patients without cartilage lesions were included. All studies had to be published in full-text in English or Norwegian after 1945.

All comparisons had to be made either at a specific follow-up point or as changes from baseline within a timeframe of 6-36 months (short term) or 3-8 years (medium term) after ACL reconstruction.

Studies reporting any type of patient-reported or objective outcome measures were considered eligible. Studies reporting on rates of return to sport, adverse events, complications and harms of intervention, reoperations, or failures of intervention were also considered eligible for inclusion.

Laboratory, cadaver, and biomechanical studies or those reporting primarily on patients with concomitant OA, bone bruises, chondromalacia, ACL revision surgery, or partial ACL tears at baseline were excluded.

### Literature Search Strategy

We collected data primarily from the following medical databases: MEDLINE via Ovid, Cochrane Library, EMBASE via OvidSP, and Web of Science. The search strategy was developed by the review team, in collaboration with a medical librarian (S.T.K.) with expertise in SR searches. A detailed draft of the final search strategy in the MEDLINE via Ovid electronic database on 09.03.2023. is displayed in **Table 1**.

**Table 1.** Literature Search Strategy in Medline via Ovid Electronic Database.

No.	Searches	Results
1	exp Anterior Cruciate Ligament Reconstruction/	7182
2	([ or anterior cruciate ligament] adj3 (reconstruction* or repair* or operation* or operative* or treatment* or surgery or surgical or surgeries)).tw, kw,kf.	15727
3	Anterior Cruciate Ligament/su [Surgery]	8752
4	Anterior Cruciate Ligament/ and (reconstruction* or repair* or operation* or operative* or treatment* or surgery or surgical or surgeries).tw,kw,kf.	9305
5	or/1-4	19077
6	[PART 1 ACL finished]	0
7	Cartilage, Articular/in [Injuries]	3750
8	Cartilage, Articular/ and (Damage* or Injur* or Defect* or Fracture* or Abrasion*).tw,kw,kf.	10348
9	([ or Chondral or Osteochondral or Hyalin] adj3 (Damage* or Injur* or Defect* or Fracture* or Abrasion*)).tw, kw,kf.	16314
10	or/7-9	21013
11	[does cartilage need specification? MeSH uses articulare]	0
12	[PART 2 ACL finished]	0
13	TreatmentOutcome/	1138049
14	Patient Health Questionnaire/	907
15	critical care outcomes/ or lysholm knee score/or patient reported outcome measures/	13554
16	(Function or treatment outcome or outcome patient reported or patient reported outcome measure or IKDC or Clinical outcome or patient reported outcome or patient reported outcome measures or KOOS or Functional outcome or patient-reported outcomes or International Knee Documentation Committee or WOMAC or Patient Health Questionnaire or Subjective Knee Evaluation Form or ARS or Oxford Knee Score or Lysholm Knee Scoring Scale or Activity Rating Scale or Marx Activity Rating Scale or Tegner activity scale or Cincinnati Knee Rating System).tw,kw,kf.	2546995
17	or/13-16	3535151
18	[PART 3. Outcome END]	0
19	General Surgery/ or Transplants/ or Autografts/ or Allografts/ or Stem Cells/ or Mesenchymal Stem Cells/	175791
20	(repair or debridement or surgery or surgical or reconstruction or operative or restoration or therapy or grafting or transplant or transplantation or treatment or augmentation or induction or resurfacing or microfracture or microdrilling or nanodrilling or drilling or Pridie or marrow stimulation or abrasion or mosaicplasty or OATS or Mega OATS or OsteochondralAutograft Transplantation Surgery or Autograft or Allograft or Cell based or ACI or Autologous chondrocyte implantation or MACI or Stem Cell or Mesenchymal stem cell* or Scaffold or AMIC or Autologous Matrix-Induced Chondrogenesis or Autogen or Autologous or Allogen).tw,kw,kf.	8965505
21	19 or 20	9013318
22	17 or 21	10900859
23	5 and 10 and 22	911

Database(s): **Ovid MEDLINE(R) ALL** 1946 to March 09, 2023 Search Strategy.

Secondary data were collected after a manual search on the topic in 3 of the most frequently cited journals,^28^ in relevant on-going or recently completed trials^29^ and snowball search of references within included studies and excluded relevant SRs found during the primary search.

### Screening across Inclusion Criteria

References obtained from the primary literature search were imported into the DistillerSR® software,^30^ where they were screened for eligibility across the predefined inclusion and exclusion criteria. Duplicate references were identified and removed. The remaining references underwent title, abstract, and full-text screening. References consistent with the inclusion criteria on every screening level proceeded to the data extraction stage. Relevant information was extracted using one of two customized, pre-designed extraction forms comprising 147 and 91 questions, respectively, in either a multiple-choice or an open-ended form. The assessment on each level was performed independently by two members of the research team. Conflicts were resolved through discussion or involvement of other members of the research team.

### Data Items

The following data items were extracted from each study eligible for qualitative or quantitative analysis: general information, study characteristics, participant characteristics, characteristics of comparator or intervention group, follow-up times, and primary and secondary outcome measures.

### Risk of Bias and Quality of Evidence Assessment

We evaluated the included randomized studies with the Cochrane Collaboration tool for assessing the risk of bias.^25,31^ The tool consists of multiple subdomains each graded with high, low, or unclear risk of bias after further qualitative elaboration.

Bias in non-randomized studies was evaluated using the Newcastle Ottawa Scale (NOS).^32^ The scale comprises 3 main domains: selection, comparability, and outcome, further divided into 2-4 separately graded subdomains. The overall score is determined as good, fair, or poor based on the section sub-scores.^32^ In the adequacy of the follow-up subdomain of NOS, the threshold for low risk of bias grading was set to a minimum of 80% of the baseline population followed up, or if more than 20% was missing, a detailed reporting of characteristics of those patients.

Two reviewers conducted the bias assessment phase, with disagreements resolved through discussion, or involvement of a third reviewer. Graphic representations of the assessments were created using the RevMan 5.4 software^33^ for randomized studies, or the robvis tool^34^ for non-randomized studies.

The quality of the results was assessed through the application of the GRADE approach^35^ and presented the results in a table format, using the GRADEpro GDT software.^36^

### Summary Measures and Planned Methods of Analysis

#### Statistical analysis

Meta-analysis was performed with the RevMan 5.4 software,^33^ pooling compatible studies reporting PROMs as continuous variables. The data were presented as pooled standardized mean differences (psmd) with 95% confidence intervals (CIs). Within-scale differences were handled by standardizing reported mean differences.

A random effects model with inverse variance weighted average was used. Heterogeneity between studies was examined with the I^2^ statistic, describing the ratio of variance in the true effects to the total observed variance.^37^ When multiple interventional groups were present,^38-40^ single interventional groups were created by pooling patient data and calculating combined means and standard deviations (SDs) for every intervention group.

When multiple studies reported results from the same cohort, only one study using a single outcome measure in a designated timeframe was used. The process was performed according to the criteria and recommendations of the Cochrane Handbook for Systematic Review of Interventions.^25^

Inter-rater agreement of bias assessment was analyzed with the Kappa score and was interpreted on a range from no agreement to almost perfect agreement according to recommended guidelines.^41^

## Results

### Study Selection

**Figure 1.**
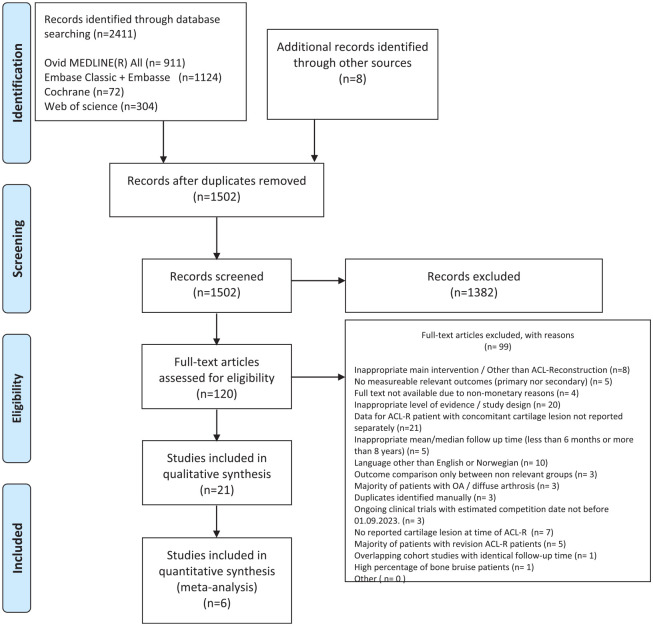
The PRISMA flow diagram represents the search and selection procedure applied during the literature review.

Our main search identified 2411 studies, and manual search another 8. Among the 120 initially relevant studies, 21 were included in the SR, while 6 were used for meta-analyses. The complete selection procedure, with detailed reasons for exclusion, is presented in **Figure 1.**

### Study Characteristics

All 21 studies were published in English. Among them were 15 “registry based” or “prognostic (prospective) cohort” studies^39,40,42-54^; 2 “interventional non-randomized”^55,56^; 1 “interventional randomized”^57^; 1 “prospective (observational) comparative”^58^; 1 “exploratory”^59^; and 1 “natural history”^38^ study. A complete overview of the study characteristics is presented in **Tables 2 and 3.**

**Table 2.** Study and Patient Characteristics.

Study characteristics											
(I=Intervention group=YES cartilage lesion; C=Control Group=NO cartilage lesion)											
Source	Study Design	Number of Patients at Follow-Up or Baseline	Mean/Median Age	Gender	Cartilage Lesion Characteristics	Concomitant Cartilage Procedures	Follow-Up Time and Rate-Primary Outcomes	Follow-Up Time and Rate—Secondary Outcomes	Outcome Measures Used	Main Results at Follow-Up	Main Takeaways
Barenius *et al.*^53^	Not specified (Registry-based study)	8,584 at baseline and 3,556 at follow-up	Age subgrouped into: <18 years = 751 (21%) 18-34=1,951 (54.9%) 35-54 =825 (23.2%) >55 29 (0.8%)	Male: 1,825 (51.3%) and Female 1,731 (48.7%)	Total number of lesions 884 (24.9%). Location, grade, and size are not reported	Not reported	X	2-year mean follow-up. Total of 3,556 (41.4%) patients followed up	Binary categorical outcome of functional recovery (FR) or treatment failure (TF) based on KOOS scores at 2-year follow-up	Relative risk (RR) of presence of cartilage lesion for FR is 0.80. For TF RR is 1.04 (n.s.). Univariable regression using cartilage lesion as exposure: for FR as outcome odds ratio is 0.76 (*p* = 0.01). For TF as outcome odds ratio is 1.06 (n.s.). Multivariable model with cartilage lesion as exposure for FR as the outcome was also implemented, with the result being unreported but also nonsignificant	Presence of cartilage lesion at the time of ACL-R is a negative predictor for FR in univariable regression and decreases relative risk for reaching FR threshold based on 2-year KOOS results
Tahami *et al.*^55^	Interventional study (non-randomized, prospective)	30 total at baseline. 15 C and 15 I	Mean 27.4 in the control group and Mean 29.2 in the intervention group	Male 14 (93.3%) in C and 13(86.7%) in I. Female 2(13.3%) in C group and 1 (6.7%) in I.	MFC 12 (80%) LFC 3 (20%). Outerbridge LVL.3 12(80%) LVL.4 3(20%). All lesions were < 2 cm^2^	Microfracture or drilling method for 15 patients in the intervention group	Mean 1 year. 30/30 (100%) patients followed up	X	Lysholm Knee Scoring Scale	Mean Lysholm scores: Mean difference between intervention group versus control group = −8.1 (n.s)	No significant differences between intervention and control group in mean Lysholm scores at 1 yr
Shelbourne *et al.*^38^	Natural history study (prospective observational)	Baseline total of 250: 125 I and 125 C	Mean 26.3 I and matched age for controls	Male 101 in I and 101 in C; Female 24 in I and 24 in C	MFC 60 (48%) LFC 65 (53%); All lesions Gr 3 or 4 by Outerbridge scale; Mean lesion size 1.7 cm^2^	No treatment for 125 lesions (100%)	Mean 8.7 years for I and mean 7.8 for C . Patients at follow-up 202 (out of 250)	Mean 6.3 years for I and 6.6 for C. 104/250 patients at follow up	Modified Noyes questionnaire; IKDC radiographic grading; strength and functional tests;	Mean Modified Noyes score for MFC 94.0 I versus 95.2 C (p=0.04); LFC 92.8 I versus 95.9 C(p<0.01); No significant difference between control and intervention group neither in stability, ROM, strength nor in IKDC radiographic assessment	Patients with an Outerbridge Grade 3 or 4 articular cartilage defect observed at the time of ACL reconstruction had significantly lower subjective scores than did a control group of patients with no defect. No significant difference in objective outcomes
Røtterud *et al.*^39^	Cohort study (prognosis)	15,783 at baseline. 8,476 at follow-up; 6,229 controls; 2,247 intervention group	Mean age 30.4; Median age 26 (9-69)	Male 4,388 (52%); Female 4,088 (48%)	688 patellar lesions (617 partial and 71 full); 213 trochlear lesions (171 partial and 42 full); MFC lesions total 1,468 (1,131 partial and 337 full); LFC 487 total (404 partial and 83 full); MTP 542 (475 partial and 67 full); LTP 550 total (493 partial and 57 full thickness). All grading according to ICRS scale, partial assuming Grade 1 or 2 and full thickness assuming Gr 3 or 4. A total of 2,283 lesions were less than 2 cm^2^ and 1,511 were equal or more. Size was not reported for 199 lesions	Treated (508); Untreated (2,732) Unreported lesions (708)	Mean follow-up 2.1 years. Total 8,476 (54%)	X	KOOS	At 2-year follow-up, **mean crude KOOS subscale scores** for ACL-R+No cartilage lesion were: KOOS pain 85.1 (84.7-85.5); KOOS symptoms 78.3(77.9-78.7); KOOS ADL 91.6 (91.2-91.9); KOOS sport/rec 66.4(65.7-67.0); KOOS Qol 63.9 (63.3-64.5). Mean crude KOOS subscale scores for ACL-R+ Partial thickness lesions were KOOS pain 84.5 (83.8-85.3); KOOS symptoms 78.5 (77.6-79.3); KOOS ADL 90.5 (89.8-91.2); KOOS sport/rec 64.1 (62.7-65.5); KOOS Qol 62.7 (61.6-63.9). Mean crude KOOS subscale scores for ACL-R+Full-thickness cartilage lesion were KOOS pain 81.3 (79.7-82.9); KOOS symptoms 75.0 (73.4-76.6); KOOS ADL 87.7 (86.3-89.1); KOOS sport/rec 58.1 (55.6-60.6); KOOS Qol 58.2 (56.1-60.2). **Partial thickness cartilage lesion as predictor of 2-year KOOS scores:** Adjusted regression with ACL-R+No cartilage lesion as reference group: KOOS pain Beta coeff. –0.2 KOOS symptoms Beta coeff. –0.3; KOOS ADL Beta coeff. –0.3; KOOS Sport/Rec Beta coeff. –1.1; KOOS QoL Beta coeff. –1.1. All values were nonsignificant. **Full (Gr 3/4) thickness cartilage lesion as a predictor of 2-year KOOS: Adjusted regression with ACL-R+ No cartilage lesion used as reference group**; KOOS Pain Beta coeff. –3.3; KOOS symptoms Beta coeff. –4.8; KOOS ADL Beta coeff. –2.6; KOOS Sport/Rec Beta coeff. –6.6; KOOS QoL Beta coeff. –6.3. All values are statistically significant (*p*<0.01)	Patients with concomitant full-thickness cartilage lesions reported worse outcomes in all of the KOOS subscales compared with patients without cartilage lesions 2 years after ACL reconstruction. Partial thickness cartilage lesions did not impair patient-reported outcomes 2 years after ACL reconstruction
Gaweda *et al.*^56^	Interventional study (non-randomized, prospective)	53 total: 21 I and 32 C	Mean age 30.87 I and 33.12 C	Male 19 in I and 22 in C. Female 2 in I and 10 in C	MFC 20 lesions, LFC 2 lesions. All are graded as 3 or 4 on the ICRS scale. Mean size 1.52 cm^2^	22 OATS	Mean 17.1 months for I and 19.1 for C. No information on number of patients	Mean 17.1 months for I and 19.1 for C. No information on number of patients	Lysholm and Guillquist score; Functional Marshall score; Adverse effects; Graft failures; Reoperations; Harms of intervention	At 12 months: Mean Lysholm Control was 93.84±2.87 (*p*<0.001) versus Interventional group mean score of 89.19 (*p*<0.001). Mean difference in Lysholm score from baseline to follow-up was 31.65±6.96 for Control group and 30.66±7.79 for Intervention group, with no statistically significant difference in mean gain between the groups. Mean Marshal at follow-up: Control: 44.81±2.4 versus 43.24±1.79 Intervention group. Change in Mean Marshall in the Control group + 10.71±3.43 versus + 9.05±3.81 in the Intervention group. Difference was statistically significant (*p*=0.49—probably a mistake in the original paper). Intervention group up to 12 months: 3 patients complained about prolonged minor pain in operated knee. Up to 6 months: frequent reports of effusions, in 5 cases re-occurring in period up to 18 months post-op. No infections in this group. Control group: 1 reoperation within 6 months; 2 patients with re-occurring effusion episodes, 2 cases of infection within 1 year	Recovery and return to normal functional activity (and the patient’s satisfaction) during the first year after operation among those treated with one-stage ACL reconstruction and osteochondral grafting is slower than in those treated with isolated ACL repair
Filbay *et al.*^59^	Exploratory analysis study on prospective RCT (Kanon trial)	62 total at baseline	Mean 27; Range 18-35	Male 47 (80%); Female 15 (20%)	Patients with osteochondral lesion: 40. Location, grade, and size are not reported. Considering osseous involvement, we assume full-thickness lesions	Not reported	5 years; 59 patients (98%)	X	KOOS	A multivariable adjusted model exploring baseline osteochondral lesion as exposure and KOOS as outcome: KOOS pain effect –5.4 (95% CI –11.7 to 1.0) (n.s.); KOOS symptoms effect = 0.4 (95% CI –9.2 to 9.9) (n.s.); KOOS sport/rec effect –3.7 (–16.5 to 9.1) (n.s.); KOOS QoL effect = −12.3 (–24.2 to −0.4) (*p*=0.04)	Presence of concomitant osteochondral lesion significantly affects 5-year KOOS Qol scores in ACL-reconstructed patients
Spindler *et al.*^42^	Prognostic study	314 total at baseline, 217 at follow-up	Mean age 27 (±9.5)	Male 119 (55%) Female 98 (45%)	Total of 156 lesions Grade 2 or above. Lesions Grade 1 were not reported. Patellar lesions: 25 Gr 2/3 and 0 Gr 4; Trochlear lesions 9 Gr 2/3 and 0 Gr 4; MFC lesions 46 Gr 2/3 and 2 Gr 4; LFC lesions 42 Gr 2/3 and 4 Gr 4; MTP lesions 5 Gr 2/3 and 2 Gr 4 and LTP lesions Lat 29 Gr 2/3 and 2 Gr 4, Graded by Outerbridge scale. Lesions size not reported	Not reported	Mean follow-up 5.4±1.7 years. Patients followed up 217 (69.1%)	X	IKDC score; KOOS score; Lysholm Knee Scoring Scale	Chondromalacia of the MTP was a significant predictor of the KOOS pain subscale score (*p* = 0.01). However, chondromalacia of the patellofemoral, femoral, and/or tibial articular cartilage were not determined as significant predictors of 5-year PROMS	Presence or absence and degree of chondral injury were not found to be significant predictors of future function
Ulstein *et al.*^40^	Cohort study	15,783 at baseline and 8,470 at follow-up	Median 27; Range 9-69	Female 4,125 (49%); Male 4,355 (51%)	Partial thickness lesions: Patella 393(19%); Trochlea 149(5); MFC1,099(39%); LFC356(13%); MTP411(14%); and LTP417(15%) Total: 1,048<2 cm^2^<573 Full-thickness lesions: Patella 67(10%); Trochlea 45(7%); MFC 344(52%); LFC 82(13%); MTP 66(10%); LTP 52(8%) Total 310<2 cm^2^<248	No treatment, debridement, microfracture or other methods	5.1 years: 8,470 (56%)	X	KOOS score	Crude mean KOOS: KOOS pain No lesion: 86.2 (85.8-86.6); Partial thickness 85.3(84.5-86.1): Full thickness 79.9 (78.0-81.4);: KOOS symptoms No lesion: 79.7 (79.2-80.1); Partial thickness 79.3(78.4-80.2): Full thickness 74.2(72.5-75.6):; KOOS ADL No lesion: 92.1 (91.7-92.4); Partial thickness 90.6(89.8-91.3): Full thickness 86.0(84.4-87.5):; KOOS Sport No lesion: 70.6 (69.9-71.2); Partial thickness 68.4(67.1-69.6); Full thickness 61.6(59.2-64.0); KOOS QoL No lesion: 67.2 (66.6-67.8); Partial thickness 66.3(65.1-67.4): Full thickness 60.0 (57.8-62.2) Adjusted multivariable regression beta coefficient with no lesion as baseline: Partial thickness KOOS pain –0.8: KOOS symptoms –1.1; KOOS ADL –0.7; KOOS sport –1.8; KOOS Qol –1.5 (all n.s.):; Full-thickness beta coefficients KOOS pain –6.0: KOOS symptoms –6.5; KOOS ADL – 5.9; KOOS sport –8.1; KOOS Qol –8.0 (*p*<0.001)	ACL-injured patients with concomitant full-thickness cartilage lesions reported worse outcomes and less improvement than those without cartilage lesions 5 years after ACL-R
Cox *et al.*^43^	Prognostic cohort study	1,512 total	Median age 23	Male 785 (56%); Female 626 (44%)	1,289 lesions reported: Patellar lesions 281(20%); Trochlear 124(9%); MFC 352(25%) LFC 277(20%): MTP 81(6%) LTP 174(12%). Out of total number of lesions 133 Gr 1, 747 Gr 2; 325 Gr 3 and 75 Gr 4 by Outerbridge scale	No treatment 1,010; Chondroplasty 487; Microfracture 24; arthroplasty 8: Mosaicplasty 5; Other procedures 2	2 years; 1,308 (87%) patients and 6 years; 1,307 (86%) patients	X	IKDC score; KOOS score; Marx activity rating scale	Significant predictors of PROMS at 6-year follow-up, reported as Odds ratios with 95% CI were: 1. For KOOS symptoms: Cartilage damage at LFC Normal/Grade 1 versus Gr 4 0.52 (0.28-0.97) (*p*=0.03) and MTP Gr 2 versus Gr 3/4 OR=0.30(0.12-0.80) (*p*=0.03) 2. For KOOS ADL: Cartilage damage at trochlea normal/Gr 1 versus Gr 3/4 OR=0.49(0.28-0.85) (*p*=0.032) 3. For KOOS Sport/Rec: Cartilage damage on MTP Gr 2 versus Gr 3/4 OR=0.39 (0.19-0.78) (*p*=0.018) 4. For IKDC MTP lesion Grade 1 versus Gr 3/4 OR=0.29(0.12-0.63)	Articular cartilage injury at the time of ACL-R is a significant predictor of IKDC and KOOS scores 6 years after ACL-R. Having a Grade 4 MFC lesion significantly reduced patient’s Marx activity level score at 6 years
Røtterud *et al.*^44^	Prognostic; prospective cohort study	30 Intervention group and 59 control group patients at baseline	Mean age (SD) at follow-up: Intervention group = 30.4 (7.0) years and control group 30.9 (6.9) years	At follow-up: number of male patients: Intervention group =70% (of 30); Control group 70% (of 50). Number of female patients was: intervention group = 30% (of 30); and control group = 30% (of 50)	30 lesions total. Patellofemoral compartment 4(13%), MFC and MTP total 20 (67%), LFC and LTP total 6 (20%). All lesions graded as Grade 3 or 4 according to ICRS scale. Size of lesions: ≤2 cm^2^ in surface = 22 (73 %) and > than 2 cm^2^ = 8 (27%)	23 untreated lesions, 4 debridements (with removal of unstable cartilage), and 3 microfractures	*Excluded from study—subcohort of larger study	Median follow-up is 2.1 (2-5) years. Also reported mean follow-up with (SD): For intervention group 2.7 years (1.1) and for control group 2.7 years (1.1). Follow-up is 30/30 (100%) for intervention group and 50/59 for control group	*Primary outcomes excluded—subcohort of larger study. Secondary outcomes are reoperations and additional knee injuries sustained during follow-up period	At follow-up: 6 surgeries in different patients during follow-up time: 1. Diagnostic arthroscopy: rupture of ACL graft 2. Removal of scar tissue 3. Synovectomy 4. Meniscus surgery 5. ACL revision reconstruction 6. ACL revision reconstruction in control group. In contrast 5 surgeries during follow-up period: 1. Diagnostic arthroscopy: findings unknown 2. Removal of cyclops formation 3. Microfracture, meniscus surgery 4. Cartilage debridement, meniscus surgery, ACL revision reconstruction 5. Microfracture, meniscus surgery, ACL revision reconstruction in intervention group	X
Jones *et al.*^45^	Not specified	1,512 cohort for primary outcomes. Within that nested cohort of 425 subjects for evaluation of secondary outcomes	Median age 23 in main cohort: Median age 20 in nested cohort	Male 785 (56%); Female 626 (44%)	Size, location and grading were not reported	Not reported	6 years: 1,308 (87%)	2 to 3 years post ACL reconstruction. Total of 262/426 participants in nested cohort	IKDC score: KOOS score: The most narrow point (least distance between femur and tibia) of medial tibiofemoral compartment joint space evident on radiograph imaging compared with contralateral normal (never operated) knee	**At 6-year predictors of IKDC:** MFC cartilage (nl/Gr 1 vs. 4) -> coeffi. 0.45. LFC cartilage (nl/Gr 1 vs. 3) coeff. 0.60 LFC (nl/Grade 1 vs. 4) coeff. 0.40 LFC cartilage (nl/Grade 1 vs. 3) coeff. 0.64 LFC (nl/Grade 1 vs. 4) coeff. 0.51 MTP cartilage (Gr 2 vs. 3 or 4) coeff. 0.3 **KOOS Pain score predictors** MFC cartilage (nl/Grade 1 vs. 4) -> coeff. 0.46. MTP cartilage (Gr 2 vs. 3 or 4) coeff. 0.39; LTP cartilage (Gr 1 vs. 3 or 4) coeff. 1.47 (Gr 2 vs. 3 or 4) coeff. 0.48 Trochlea (Gr 1 vs. 3 or 4) coeff. 0.49 MFC (Gr 1 vs. 2) coefficient is 1.36 **Predictors of KOOS QoL** MFC cartilage (Gr 1 vs. 4) coeff. 0.40. LFC (Gr 1 vs. 4) coeff. 0.42; MTP cartilage (Gr 2 vs. 3 or 4) coeff. 0.39 // **Predictors of Marx activity score** MFC cartilage (Gr 1 vs. 4) -> coeff. 0.47. Medial femoral condyle articular cartilage status was not a significant predictor for **difference in medial compartment minimum joint space** width in ACL-reconstructed patients	Factors associated with worse PROM’s at 2 and 6 years include chondral injury among others. Chondral injury is not predictor for joint space narrowing at 2-year post ACLR (n.s.)
McAllister *et al.*^46^	Not stated	69 total at baseline; 55 at follow-up	Mean age 28.7 (14-53 range)	Male 30 (55%); Female 25 (45%)	Patellar chondromalacia total 15 (Gr 1 = 2, Gr 2=8, Gr 3=5); Trochlear chondromalacia 5 (Gr 1= 2, Gr 2= 2, Gr 3=1); MFC chondromalacia 15 (Gr 1=3, Gr 2=6, Gr 3=3, Gr 4=2) + 1 cartilage fracture; LFC chondromalacia 9 (Gr 1=2, Gr 2=3, Gr 3=3, Gr 4=1); MTP chondromalacia 5 (Gr 1=1, Gr 2=3, Gr 3=1) and LTP chondromalacia 8 (Gr 1=1, Gr 2=3, Gr 3=4) graded by Outerbridge scale. No information on size of lesions	No information	Mean 3.6 years; 55/69 (80%) patients	X	Physical function scores, bodily pain, and physical component summary score of SF-36	Statistically significant results of multivariate linear regression. Patellofemoral cartilage damage grade (*p* = 0.09) 95% CI = −8.4 to −2.6 Medial compartment grade *p*=0.05 95% CI = −7.2 to −1.4 were significant predictors of lower physical function assessment score. Medial compartment grade (*p* = 0.007) 95% CI = −8.6 to −1.4 were significant predictors of lower bodily pain assessment score. Patellofemoral cartilage damage grade (*p* = 0.041) 95% CI = −3.1 to −0.1; Medial compartment grade (*p*=0.33) 95% CI = −3.1 to −0.1 were significant predictors of lower physical component score of SF-36	Intra-articular injury was identified as predictor of worse outcome on SF-36; physical function, bodily pain, and physical component scores. Grade of articular cartilage damage to the patellofemoral and medial compartments was predictive of poorer physical function and physical component summary scores. Higher grade damage to the medial compartment and the presence of a lateral meniscus tear were predictive of lower bodily pain scores
Kocher *et al.*^47^	Prospective cohort study	201	Mean age 28.6 (14.4-60 range)	Male 114(57%); Female 87(43%)	Patellar *arthrosis 27 lesions; trochlear 25 lesions; MFC 45 lesions; LFC 18 lesions; MTP 18 lesions; LTP 26 lesions. Grade 1 =33; Gr 2 =49; Gr 3=39; Gr 4=38. Size not reported	Not reported	mean 35.9 months (range 24 to 87 months)	X	Degree of patient satisfaction by the outcome	No significant associations were detected between patient satisfaction with the outcome and grade of arthrosis of the medial femoral condyle (*p*=0.34), lateral femoral condyle (*p*=0.246), medial tibial plateau (*p*=0.683), lateral tibial plateau (*p*=0.055), patella (*p*=0.187), or trochlear groove (*p* =1.22)	The extent of arthrosis in the medial, lateral, and patellofemoral compartments did not have a significant relationship with patient satisfaction, suggesting that patients may be satisfied after reconstruction despite the presence of some osteoarthritis
Andernord *et al.*^48^	Prospective cohort study	13,102	Median age 25 (range 7-67)	Male 7,561 (58%); Female 5,541 (42%)	3,616 patients with at least 1 lesion. Number, grade, and size of lesions were not reported	52 (1%) had surgical treatment of cartilage lesion	X	2 years since ACLR (or until revision ACLR if that happens sooner)	Acl revision surgeries	Patients with cartilage injury at index reconstruction who received an HT autograft were less likely to undergo revision surgery compared with patients with no cartilage injuries (1.10% vs. 1.86%; RR = 0.59; 95% CI, 0.41-0.84; *P* = .004). This difference was present regardless of whether cartilage surgery had been performed (*P* = 0.515). Patients with cartilage injury at index reconstruction who received an PT autograft were more likely to undergo revision surgery compared with patients with no cartilage injuries (RR = 1.78; 95% CI, 0.65-4.87; *P* = 0.260 but result was not statistically significant in this case)	Patients with observed cartilage injuries who received an HT autograft at index ACL-R reconstruction were less likely to undergo revision surgery
Keays *et al.*^49^	Prognostic cohort study	62	Mean age 27 (range 18-38)	Male 42; Female 18	No information	No information	X	6 years; 56 total (46 for musculoskeletal part of analysis)	Kellgren Lawrence grading system and return to sport	Tibiofemoral OA: Equality of means test determined that presence of chondral damage (chondral bone involvement) at the time of ACL-R is a significant risk factor for development of tibiofemoral OA. (r = 0.411). Again, test of equality of means showed that presence of chondral injury was a significant predictor (*p*=0.003) of patellofemoral OA development at 6-year mark in ACLR patients. Strongest predictor was higher level of chondral injury (*r*=0.75)	Chondral damage was the second strongest predictor for tibiofemoral OA in ACL-R patients. The association between patellofemoral OA and chondral damage is less statistically significant
Ulstein *et al.*^50^	Prospective cohort study	89 at baseline	Mean age intervention group 34.9 Mean age control group 34.7 years	Male 53 (71.6%) Female 21 (28.4%)	4 patellofemoral; 20MFC (67%); 6LFC (20%). All Grade 3 or 4 ICRS. Size: 22<2 cm^2^ (73%) and 8>=2 cm^2^ (23%)	No treatment 23 (77%), Debridement 4, Microfracture 3	X	Median 8.2 years (6.4-9.8) 41/89 followed up	Kellgren Lawrence system and reoperations	Kellgren Lawrence score: Knees rated as equal or more than Grade 2: 12/19 in Intervention group versus 20/21 in Control group. Difference was statistically significant (*p*=0.016) Reoperations: 7 (24%) in the Intervention group versus 10 (22%) in the Control group	Radiographic knee OA of the affected knee defined as Kellgren and Lawrence ≥2 was significantly more frequent in subjects without a concomitant cartilage lesion (*p* = 0.016)
Dunn *et al.*^51^	Cohort study	446 at baseline. At follow-up 393	Median age 23 (17Q1 and 35Q3)	Male 222 (56%) Female 171 (44%)	Chondrosis total 226. Anterior chondrosis 76 (19%), medial chondrosis 77 (20%), Lateral chondrosis 73 (19%). All lesions were Gr 2 or more on modified Outerbridge scale. No information on size of lesions	No information	2 years. 393 patients (89%)	X	Marx scale	Presence of chondrosis in either department at primary ACL-R is not significant predictor of Marx score at 2-year follow-up	Condition of the articular cartilage was not predictive of activity level at 2 years

**Table 3.** Study and Patient Characteristics.

Study Characteristics ACL-R = Anterior Cruciate Ligament Reconstruction; KOOS=Knee Injury and Osteoarthritis Outcome Score; IKDC=International Knee Documentation CommiteMF=Microfracture; DB=debridement; NO TR=no treatment											
Source	Study Design	Number of Patients at Follow-Up or Baseline	Mean/Median Age	Gender	Cartilage Lesion Characteristics	Concomitant Cartilage Procedures	Follow-Up Time and Rate- Primary Outcomes	Follow-Up Time and Rate—Secondary Outcomes	Outcome Measures used	Main Results at Follow-Up	Main Takeaways
Røtterud *et al.*^52^	Cohort study (Registry based)	Baseline: 644 total ACL-R+NO TR =351 ACL-R+DB= 129 and ACL-R + MF=164	Mean age: ACL-R+NO TR =36±10 ACLR+DB=36+10 ACLR+MF=35±10	152 female	ACL-R + NO TR. Number of lesions: Patella 10(5%), trochlea 8 (4%), MFC 125(65%), LFC 28(15%), MTP 5(3%), LTP 15(8%), Undisclosed 20(10%) Depth ICRS Gr 3 =146, Gr 4=45 Size >2 cm^2^= 107, ≤2 cm^2^=82, undisclosed=2 ACL-R + DB Number of lesions: Patella 7(9%), trochlea 3 (4%), MFC 56 (72%), LFC 11(14%), MTP 1, LTP 0, Undisclosed 0: Depth ICRS Gr 3 =68, Gr 4=10: Size >2 cm^2^= 48 (62%), ≤2 cm^2^=30 (38%); ACL-R + MF Number of lesions: Patella 0, trochlea 5 (6%), MFC 73 (83%), LFC 7 (8%), MTP 2, LTP 1, Undisclosed 0: Depth ICRS Gr 3 =43 (49%) Gr 4=45 (51%): Size >2 cm^2^= 36 (41%); ≤2 cm^2^=52 (59%)	191 untreated lesions; 78 debridements; 88 microfractures	Mean follow-up 2.1 years (range 1.9-2.3) 357/644 (55%)	X	KOOS	Crude Mean KOOS scores at follow-up: ACL-R+No treatment: KOOS pain 82.2 (79.6-84.7). KOOS symptoms 75.5 (72.9-78.2). KOOS ADL =88.3 (86.0-90.5). KOOS Sport/Rec= 60.4 (56.2-64.5). KOOS QoL 59.8 (56.3-63.3). ACL-R + DB: KOOS Pain 82.2 (78.8-86.7). KOOS symptoms 77.2 (73.4-80.9). KOOS ADL 91.2 (88.2-94.2). KOOS Sport/Rec = 61.9 (55.4-68.4). KOOS QoL = 62.6 (57.3-67.9) ACL-R+MF: KOOS Pain 79.7 (76.5-83). KOOS symptoms 72.3 (68.5-76.1). KOOS ADL 86.3 (83.0-89.5). KOOS Sport/Rec = 51.5 (45.5-57.5). KOOS QoL = 51.7 (47.1-56.3) Adjusted linear regression using ACL-R+No treatment group as reference. For ACL-R+DB group: KOOS pain b=0.2; KOOS Symptoms b=1.0; KOOS ADL b=1.8; KOOS sport and rec b=−0.2; KOOS QoL b=2.1. All values non-significant Adjusted linear regression for ACL-R+MF group: KOOS pain b=−4.2 (n.s); KOOS Symptoms b=−3.3 (n.s); KOOS ADL b=−2.7(n.s); KOOS sport and rec b=−8.6 (*p*=0.032); KOOS QoL n b=−7.2 (*p*=0.028)	MF of concomitant full- thickness cartilage lesions showed adverse effects on patient-reported outcomes at 2-year follow-up after ACL reconstruction. Debridement of concomitant full-thickness cartilage lesions showed neither positive nor negative effects on patient-reported outcomes at 2-year follow-up after ACL reconstruction
Gudas *et al.*^57^	Prospective comparative study	136 total	Mean age 34.1; Age range 22-45	88 male, 48 female	ACL-R+ intact cartilage group = 34 lesions; For all groups (ACL-R+ OATS; ACL-R+DB and ACL-R +MF) all 34 lesions were located on MFC. Depth of lesion: ACL-R +OATS=Gr 3=11, Gr 4=13; ACL-R+MF Gr 3=12, Gr 4=12; ACL-R+ DB=Gr 3=14, Gr 4=10 (data for 10 lesions in each group is unreported, but it is assumed they are grade 3 to 4); Size of lesions: ACL-R+OATS= mean diameter 3.1±1.2 cm range; ACL-R+MF 2.7±3.6 mean diameter; ACL-R+DB mean diameter 2.9 ± 6.2 cm range	34 OATS: 34 Debridement: 34 Microfracture	Mean follow-up 36.1 months (range 34-37). 97/102 (95%) patients followed up	11.1 months (range 9 to 14)	IKDC score, Mean Tegner score, Clinical assessment, Return to previous level of activities	Isolated ACL-R group Mean IKDC score 89 (estimated from graph). Mean Tegner 7.5. At 11.1 months (9-14) 32/34 patients from this group returned to their previous level of activity. ACL-R+OATS group mean IKDC=88 (estimated from graph). Mean Tegner at 7.1: ACL-R+MF group = Mean IKDC 85.5 (estimated from graph). Mean Tegner 6.9 ACL-R+DB Mean IKDC=84 (estimated from graph). Mean Tegner 6.2. At 11.1 months (range 9-14), in ACL-R OATS group 30/34, in ACL-R MF 28/34 and in ACL-R DB 27/34 patients returned to previous level of activity. Statistically nonsignificant difference between all 4 groups in clinical evaluation (pivot shift scores, Lachman test scores, IKDC radiograph and KOOS scores).ACL-R OATS group mean IKDC score is significantly higher than both ACL-R MF group score (*p*<0.03) and ACL-R DB score (*p*=0.018). However, mean IKDC score in ACL-R OATS group was significantly worse than score in Isolated ACL-R group (*p*=0.043). No significant difference in mean IKDC scores between ACL-R MF and ACL-R DB group (*p*=0.058). Tegner scores were significantly lower in ACL-R DB and ACL-R MF compared with ACI-R OATS and Isolated ACL- R group	All 4 groups fared significantly better at the 3- year follow-up than preoperatively. The OAT- ACL-R group’s IKDC subjective knee evaluation was significantly better than that of the MF-ACL-R group and DB-ACL-R group. However, the IKDC subjective score of the Isolated-ACL-R group was significantly better than the OAT-ACL-R group’s IKDC evaluation. There was no significant difference between the MF-ACL-R and DB-ACL-R groups’ IKDC subjective scores (*P*=0.058)
Osti *et al.*^58^	Not reported (prospective comparative)	50 total	Median age for ACL-R +MF group = 29 (19-30) Median age for ACL-R + radiofrequency ablation group = 28 (17-29)	Male 28; Female 22	ACL-R + MF group: Total lesions 25. Out of that MFC lesions 18 (72%), trochlear lesions 4 (16%), LFC lesions 2 (8%) and tibial plateau lesions 1 (4%). All lesions graded as Outerbridge level 3 or 4, with surface in range of 1-3 cm^2^. ACL-R + radiofrequency ablation. Total lesions 25. Out of that trochlear lesions 4, MFC lesions 19(76%), LFC lesions 2 (8%). All graded as level 1 or 2 according to Outerbridge scale, surface in range of 1-3 cm^2^	Microfracture 25 Radiofrequency ablation 25	2 years and 5 years	2 years and 5 years	IKDC score, Lysholm Knee Scaling Score, WOMAC Score (only at 5-year follow-up), Tegner Activity Score, Lachman and pivot shift test. Also return to sport, OA radiographic assessment using Fairbank scale and joint space narrowing assessment via Merchant view at 45 degrees	At 2-year follow-up: No significant difference in Lachman, pivot shift and KT 1,000 test between groups. Median Lysholm in ACL-R+MF group 89 versus 91 in ACL-R+RF group. IKDC: 23 ACL-R+MF group versus 24 knees in ACL -R+radiofrequency ablation group evaluated as normal or nearly normal. Median Tegner score equal between groups (7 points) (n.s.). Return to preinjury level: ACL-R+MF 21/25 versus 23/25 in ACL-R+radiofrequency group (n.s.) At 5-year follow-up: Nonsignificant difference between groups on Lachman test, pivot shift test and KT 1,000. Median Lysholm Score: ACL-R+MF group 83 versus ACL+RF 87 (n.s.) (*p*=0.07). IKDC score: ACL-R+MF 18/25 versus ACL-R+RF 23/25 knees rated as normal or near normal. Median Tegner score ACL-R+MF 6 versus ACL-R+RF 7. 9/25 patients in ACL+MF group decreased activity level versus 2/25 in ACL-R+RF group. Fairbank degenerative changes Gr 1 or 2 seen in 12/25 ACL-R+MF versus 5/25 ACL-R+RF group knees (*p*<0.05). Merchant test detected joint space narrowing in 7/25 ACL-R+MF versus 2/25 ACL-R+RF group patient (p<0.05). WOMAC pain and function index score significantly higher for ACL-R+MF versus ACL-R+RF group	Higher rates of subjective symptoms and reduction of sport activity have been shown in Grade 3–4 cartilage-injured knees, regardless of the amount of meniscus removal. For ACL-injured patients with cartilage lesion less than 2 cm^2^ in diameter, microfracture leads to good short and midterm improvement, with great percentage return to preoperative sport activity. No data is available if microfracture can prevent degenerative changes in the long term, but it does not seem to be the case
Ulstein *et al.*^54^	Cohort study (Registry based)	644 at baseline and 351 at follow-up	Mean age: 41.2±10.4 years	In no treatment group: Male 107 (53%) and female 96 (47%). In ACL-R Debridement group: Male 44 (63%) and female 26 (37%). In ACL-R+ Microfracture group: Male 49 (52%) and female 46 (48%)	ACL-R + No treatment: Patella lesions=11 (5%), Trochlea=16 (8%), MFC=128 (63%), LFC 27(13%) MTP=6(3%) and LTP=15 (7%). There were 42 lesions (21%) graded 4 on ICRS scale. Total of 94 (46%) were below, and total of 107(53%) were equal or greater than 2 cm^2^ in surface. Size was not reported for 2 lesions. ACL-R + DB: Patella lesions=6 (9%), Trochlea=3 (4%), MFC=53 (76%), LFC 7(10%) MTP=1(1%) and LTP=0(0%). There were 8 lesions (11%) graded 4 on ICRS scale. Total of 24 (34%) were below, and total of 46 (66%) were equal or greater than 2 cm^2^ in surface. ACL-R + MF: Patella lesions=1 (1%), Trochlea=4 (4%), MFC=78 (82%), LFC 7(7%) MTP= 3 (3%) and LTP=2 (2%)	No treatment 203 Debridement 70 Microfracture 95	Mean follow-up 5.1±0.1 years Patients at follow-up 368/644 (57%)	X	KOOS subscales	Crude Mean KOOS scores at follow-up: ACL-R+No treatment: KOOS pain 81.5 (79.0- 84.1). KOOS symptoms 75.1 (72.4-77.9). KOOS ADL =87.5 (85.1-89.6). KOOS Sport/Rec= 63.2 (59.2-67.3). KOOS QoL 61.6 (58.0-65.1). Crude Mean KOOS scores at follow-up: ACL-R+ Debridement: KOOS pain 82.1 (77.7-86.5). KOOS symptoms 78.0 (73.0-83.0). KOOS ADL =89.5 (85.5-93.5). KOOS Sport/Rec= 68.2 (62.0-74.5). KOOS QoL 65.7 (58.9-72.6). Crude Mean KOOS scores at follow-up: ACL-R+ Microfracture: KOOS pain 78.5 (74.6-82.5). KOOS symptoms 73.4 (69.6-77.2). KOOS ADL =85.2 (81.4-88.9). KOOS Sport/Rec= 57.5 (51.9-63.0). KOOS QoL 55.6 (50.8-6.4). Adjusted linear regression using ACL-R+No treatment as reference group: Results for ACL-R+ Debridement group: KOOS pain beta –1.0; KOOS symptoms beta 2.2; KOOS ADL beta 0.3: KOOS sport/rec beta 2.9; KOOS QoL beta 1.8. Adjusted linear regression using ACL-R+No treatment as reference group: Results for ACL-R+ Microfracture group: KOOS pain beta –1.7; KOOS symptoms beta 0.3; KOOS ADL beta –1.8: KOOS sport/rec beta –5.0; KOOS QoL beta –5.7. For both groups, neither value reached statistical significance	Compared with leaving concomitant full-thickness cartilage lesions untreated at the time of ACL-R, debridement and MF showed no effect on patient-reported outcomes 5 years after surgery

### Primary Outcomes

Of 21 included studies, 14 compared PROMs in patients with and without cartilage lesions, from 6 months to 8 years after ACL reconstruction.^38-40,42,43,45-47,51,53,55-57,59^ A total of 14,311 individuals (46.9% females, 9-69 years old at baseline) were enrolled, with 9,123 reported lesions. Most studies reported cartilage lesions independently of grade^39,43,46,47^ while others only reported full-thickness (at least Grade 3 or higher) lesions^59^ according to Outerbridge^38,55^ or International Cartilage Repair Society classification (ICRS).^56,57^ One prognostic study^42^ reported Outerbridge Grade 2 or higher lesions, but not Grade 1 lesions. Cartilage lesions treatment varied between studies: from surgically untreated^38,39,43,57^ to microfracture,^43,55,57^ chondroplasty (debridement),^43,57^ abrasion arthroplasty,^43^ mosaicplasty,^43^ drilling,^55^ and Osteochondral Autologous Transplantation Surgery (OATS).^56,57^

### Quantitative Analysis of Primary Outcomes

Six studies with 8,789 patients (38% with CFCLs) were eligible for quantitative analysis. We obtained data from a range of different outcome assessment systems.

#### Cumulative short- and medium-term follow-up

**Figure 2.**
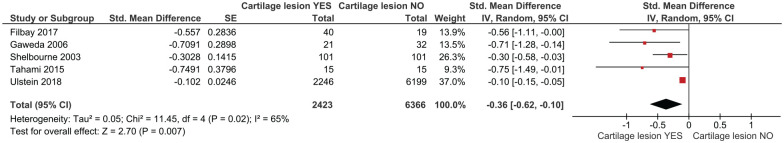
Cumulative short- to medium-term PROMs.

**Figure 3.**
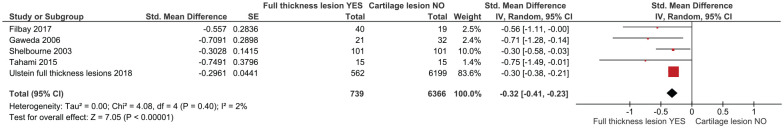
Cumulative short- to medium-term PROMs (full-thickness cartilage lesions only).

As our primary objective: In the cumulative short- to midterm, regardless of grading, cartilage lesions were associated with worse PROMS (**Fig. 2**). Considering only full-thickness cartilage lesions, we found a similar effect size, with narrower CI and very low heterogeneity (**Fig. 3**).

#### Short-term follow-up (6-36 months)

**Figure 4.**
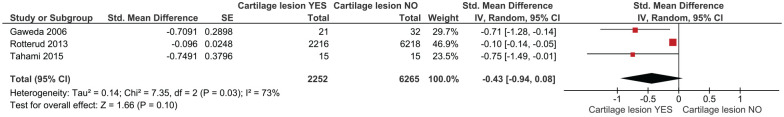
Short-term PROMs.

**Figure 5.**
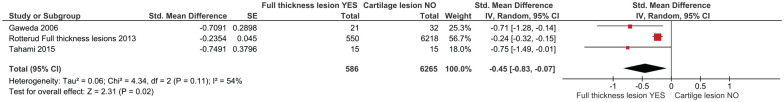
Short-term PROMs (full-thickness cartilage lesions only).

Concerning short-term follow-up,^39,55,56^ there is some evidence associating cartilage lesions with worse PROMS, but with high heterogeneity, and no statistical significance (**Fig. 4**). Full-thickness cartilage lesions (*n* = 586) are a significant negative predictor of short-term PROMS, with acceptable heterogeneity (**Fig. 5**).

#### Medium-term follow-up (3-8 years)

**Figure 6.**
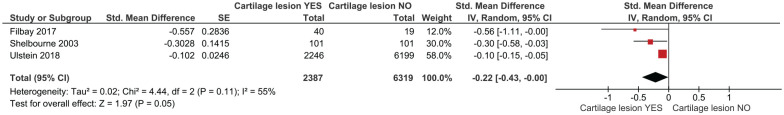
Medium-term PROMs.

**Figure 7.**
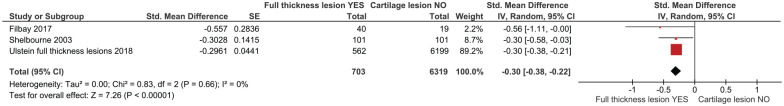
Medium-term PROMs (full-thickness cartilage lesions only).

At midterm follow-up,^38,40,59^ cartilage lesions predicted worse PROMs regardless of grading (**Fig. 6**). The effect was statistically significant for full-thickness lesions (**Fig. 7**).

## Qualitative Description

### Primary Outcomes (PROMs)

For the remaining 8 studies^42,43,45-47,51,53,57^ investigating PROMS that could not be included into the quantitative analyses, a detailed narrative summary of results was performed.

Three studies^43,45,51^ were performed on the Multicenter Orthopedic Outcomes Network (MOON) cohort. Jones *et al*.^45^ with 1,308 patients found a significant negative impact of medial femoral condyle (MFC), lateral femoral condyle (LFC), and medial tibial plateau (MTP) full-thickness (Grade 3 or 4) cartilage lesions on 6-year IKDC scores. Full-thickness cartilage lesions in the MFC, trochlea, and MTP had a significant negative impact on the 6-year KOOS pain scores. Grade 2 MFC lesions predicted higher KOOS pain scores than Grade 1 lesions in these compartments. MFC, LFC, and MTP high-grade lesions were significant negative predictors of 6-year KOOS Qol score. Cox *et al*.^43^ also found a significant negative impact of high-grade lesions of LFC and MTP on KOOS symptoms score, trochlear lesions on KOOS ADL score, and MTP high-grade lesions on KOOS sport/rec and IKDC scores. As in Jones *et al*.^45^ a significant negative effect of Grade 4 MFC lesions on the Marx activity score at 6-year follow-up was observed, which contradicts the findings in Dunn *et al*.^51^ (393 patients), where no significant associations between location and the grading of cartilage lesions and the 2-year Marx scores were reported.

Spindler *et al*.^42^ found a significant negative correlation (*p* < 0.001) between the presence of MTP chondromalacia and 5-year KOOS pain score, while other PROMs were not significantly affected by location or degree of cartilage lesion.

One prospective cohort study^47^ across 201 subjects reported the degree of patient satisfaction at a mean follow-up of 35.9 months. No statistically significant associations were found between the primary outcome scores and cartilage lesions, regardless of grading.

In a study by Barenius *et al*.^53^ cartilage lesions at the time of ACL reconstruction were significantly associated with a decreased likelihood (RR=0.80, OR=0.76) of functional recovery at 2-year mean follow-up.

In a study by Gudas *et al*.^57^ containing 136 patients at baseline, with a mean follow-up of 3 years, 34 patients with isolated ACL reconstruction reported significantly better IKDC subjective scores compared with the groups of patients with concomitant full-thickness cartilage lesions treated with either microfracture, debridement, or OATS. A significant difference was also observed in the median Tegner scores, favoring the isolated ACL reconstruction group over both microfracture and debridement groups.

Finally, in a study by McAllister *et al*.^46^ of 55 patients followed for a mean of 3.6 years, patellofemoral and MFC cartilage lesions were predictors of both lower physical function and physical assessment domain scores of SF-36 test, with MFC cartilage lesions also predicting inferior bodily pain assessment scores. Moreover, the grading of cartilage lesions was associated with the negative effect size of the outcome.

### Secondary Outcomes

Seven studies^38,44,45,48-50,56^ examined the effect of cartilage lesions on objective outcome measures other than PROMs, in ACL-reconstructed patients at short- to midterm follow-up.

#### Post-operative OA assessment

Four studies^38,45,49,50^ across 511 patients, performed radiographic OA assessments. Two studies found no significant correlation between cartilage lesions and radiographic signs of OA, that is, MFC and LFC cartilage lesions were not associated with worse 6-year IKDC radiographic scores,^38^ and MFC cartilage lesions were not related to narrowing of the medial compartment joint space in a nested cohort of 262 patients from the MOON cohort at 2-3-year follow-up.^45^ A prognostic study by Keays *et al*.,^49^ on 56 patients with 6-year follow-up, determined increased grading of full-thickness cartilage lesions with subchondral bone involvement to be a significant risk factor for both patellofemoral and tibiofemoral OA development. However, Ulstein *et al*.^50^ reached the opposite conclusions after following 41 patients for a median of 8.2 years; in the OA assessment, using the Kellgren Lawrence scale, Grade 2 or higher OA was significantly more frequent in patients without a cartilage lesion with those with (21/22 vs. 12/19).

#### Reoperation rates

Four studies examined reoperation rates.^44,48,50,56^ Since two of them^44,50^ used the same Norwegian Knee Ligament Registry (NKLR) subcohort, and the third, and largest one^48^ did not report the number of patients at follow-up, the total number of patients is hard to estimate. In Gaweda *et al*.^56^ only 2 reoperations were reported after 6 months among 32 patients without cartilage lesions, compared with none among 21 patients with cartilage lesions. In another short-term (mean 2.1 years) follow-up study by Røtterud *et al*.^44^ the reported reoperation rates were low and similar in the two groups: 6/50 (12%) without cartilage lesions, and 5/30 (17%) with. At long term (mean 8.4 years) in the same cohort, the reoperation rates remained balanced but increased to 22% and 24%.^50^ In a large cohort of 13,102 patients, those who received a hamstring tendon (HT) autograft were significantly less likely to receive revision ACL-surgery within 2 years when cartilage lesions were present.^48^ In contrast, more patients who received bone-patellar tendon-bone (BPTB) autograft underwent ACL revision surgery if a cartilage lesion was present at index surgery, but the difference was not statistically significant.

##### Comparison of surgical treatments

Outcome measures from 4 studies with altogether 518 patients treated with different surgical approaches were reported.^52,54,57,58^; 147 patients received microfracture,^52,54,57,58^ 112 surgical debridement,^52,54,57^; 34 OATS,^57^; 25 radiofrequency ablation^58^ and the rest no surgical treament.^52,54,58^ In the short term (2.1 years), microfracture significantly predicted worse KOOS scores (KOOS Sport/rec and KOOS QoL) compared with no treatment, while differences between debridement and no treatment on all KOOS subscales were not significant.^52^ In the medium term (5.1 years), KOOS subscale scores did not significantly differ between microfracture, debridement, or no treatment in the same subcohort of Swedish and Norwegian KLR 2005-2008 cohort.^54^ One prospective comparative study^58^ reported no difference in PROMs in patients treated with microfracture (*n*= 25), compared with radiofrequency ablation (*n*= 25) at 2-year follow-up, while mean Tegner scores favored radiofrequency ablation group at 5-year follow-up. A randomized study^57^ reported no difference between the mean subjective IKDC scores and the median Tegner scores for patients treated with debridement (*n*=34) and microfracture (*n*=34) at 3-year follow-up, with both treatment groups reporting significantly worse scores compared with OATS (*n*=34).

##### Risk of bias within studies

All included studies except Gudas *et al*.^57^ were non-randomized in design, and assessed for bias using the NOS. Of 13 studies reporting PROMS, 8 were graded as low^38,39,40,43,45,51,55,59^ and 5 as high risk of bias,^42,46,47,53,56^ the most common reasons being the usage of subjective outcome assessments (all studies), low rate of follow-up^42,46,53^ and underreporting of adjustment factors other than age and gender.^39,40,42,43,45-47,51,53,55,59^ (Table 4). In 7 studies examining secondary outcomes, 4 were rated as high^45,48,49,56^ and 3 as low risk of bias (Table 5).^38,44,50^

**Table 4.** Newcastle Ottawa Scale Bias Assessment-PROMs.

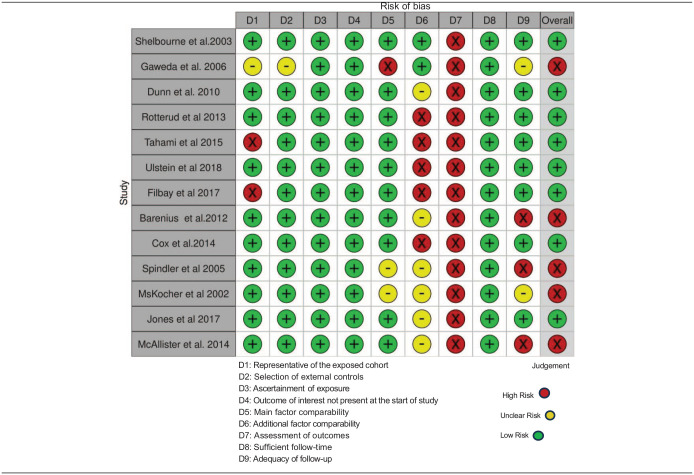

**Table 5.** Newcastle Ottawa Scale Bias Assessment-Objective Outcomes.

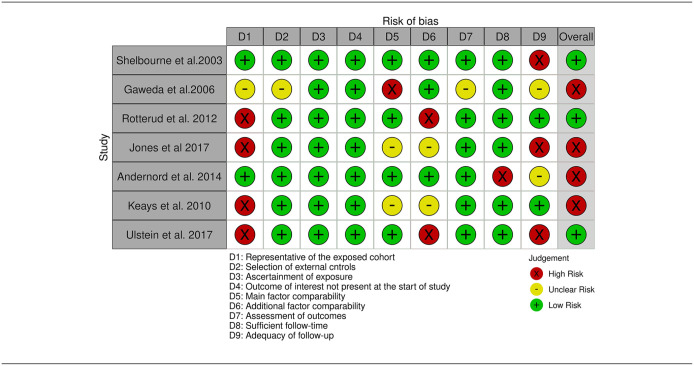

In 2 out of 3 studies comparing different cartilage procedures, the risk of bias was graded as low^52,54^ and in 1 it was rated as high^58^ due to the inability to adjust for size and depth of the cartilage lesions (Table 6). Gudas *et al*.^57^ study was graded according to the Cochrane risk-of-bias tool for randomized trials,^26^ faring poorly in the sections of sequence generation, allocation concealment, blinding of participants and personnel and selective outcome reporting, due to lack of information provided in the study (**Table 7**).

**Table 6.** Newcastle Ottawa Scale Bias Assessment-Comparison Between Different Cartilage Procedures.

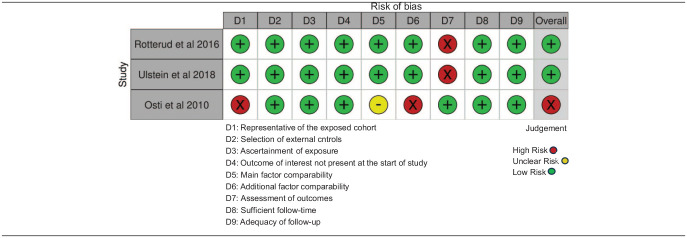

**Table 7.** Cochrane Risk of Bias Tool Bias Assessment—Randomized Study Designs.

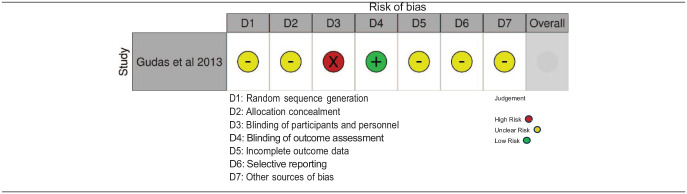

The degree of inter-rater agreement was strong (Kappa=0.843) and moderate (Kappa=0.719) for studies reporting primary and secondary outcomes, respectively.

## Discussion

### Primary outcomes

The most important finding of the present study, estimated with moderate uncertainty on the available level 1 and 2 evidence data, was that high-grade cartilage lesions in ACL-reconstructed patients are associated with worse cumulative short- to midterm PROMS. Our estimates from a similar analysis, performed separately for short- and midterm follow-up data, were of high uncertainty. Additionally, we concluded with a very high degree of uncertainty, that cartilage lesions in ACL-reconstructed patients are associated with worse short-, mid-, and cumulative short- to midterm PROMS, regardless of lesion depth (Table 8).

**Table 8.** GRADE Summary of Findings Table.

Concomitant Cartilage Lesion YES Compared With Concomitant Cartilage Lesion NO as Predictor of Various Outcomes in ACL-Reconstructed Patients. *Different cartilage Procedures as Predictors of Functional Outcomes in ACL–Reconstructed Patients													
Certainty Assessment													
Outcome Measured	Participants (Studies) Follow-Up	Risk of Bias	Inconsistency	Indirectness	Imprecision	Publication Bias	Large Effect	Plausible Confounding	Dose Response Gradient	Number of Controls	Number of Patients in Intervention Group	Pooled Magnitude of Effect (95% CI)	Overall Certainty of Evidence
Short- and midterm PROMS regardless of lesion thickness (follow-up: range 6 months to 8 years)	8,789 (5 non-randomized studies)	Not serious	Serious^a^	Not serious	Not serious	Undetected^b^	No ^c^	No	No	6,366	2,423	PSMD –0.36 (–0.62, –0.10)	⨁〇〇〇 Very low
Short- and midterm PROMS for patients with high-grade lesions (follow-up: range 6 months to 8 years)	7,104 (5 non-randomized studies)	Not serious	Not serious	Not serious	Not serious	Undetected^b^	Large^c^	No^d^	No	6,366	738	PSMD –0.32 (–0.41, –0.23)	⨁⨁⨁〇 Moderate
Short-term PROMS regardless of thickness lesion (follow-up: range 6 months to 3 years)	8,518 (3 non-randomized studies)	Serious^e^	Serious^f^	Not serious	Serious^g^	Undetected^b^	No^c^	No	No	6,265	2,253	PSMD–0.43 (–0.94, 0.08)	⨁〇〇〇 Very low
Short-term PROMS for patients with high-grade thickness lesion (follow-up: range 6 months to 3 years)	6,852 (3 non-randomized studies)	Not serious^h^	Serious^i^	Not serious	Not serious	Undetected^b^	Large^c^	No^d^	No	6,265	587	PSMD –0.45 (–0.83, –0.07)	⨁⨁〇〇 Low
Midterm PROMS for regardless of lesion thickness (follow-up: range 3 years to 8 years)	8,706 (3 non-randomized studies)	Not serious	Serious^j^	Not serious	Serious^k^	Undetected^b^	No	No	No	6,319	2,387	PSMD –0.22 (–0.43, 0.00)	⨁〇〇〇 Very low
Midterm PROMS for patients with high-grade cartilage lesions (follow-up: range 3 years to 8 years)	7,022 (3 non-randomized studies)	Not serious	Not serious	Not serious	Not serious	Undetected^b^	No	No^d^	No	6,319	703	PSMD –0.30 (–0.38, –0.22)	⨁⨁〇〇 Low
Concomitant cartilage lesion in ACL-reconstructed patients as predictor of OA development in short- to midterm (follow-up: range 6 months to 8 years)	511 (4 non-randomized studies)	Serious^l^	Very serious^m^	Serious^ø^	Serious^n^	Undetected^b^	No	No	No	X	X	X	⨁〇〇〇 Very low
Concomitant cartilage lesion in ACL-reconstructed patients as a predictor for revision surgery in short- to midterm (follow-up: range 6 months to 8 years)	(3 non-randomized studies)	Serious^o^	Not serious	Not serious	Extremely serious^p^	Undetected^b^	No	No	No	X	X	X	⨁〇〇〇 Very low
*Microfracture versus no treatment at short term (follow-up: range 6 months to 3 years)	279(1 non-randomized study)	Not serious	Not serious^q^	Not serious	Serious^r^	Undetected^b^	Large^s^	No	No	88	191	Adjusted beta coeff. –7.2 (–13.6 to −0.8)	⨁⨁〇〇 Low
*Microfracture versus no treatment at medium term (follow-up: range 3 years to 8 years)	298 (1 non-randomized study)	Not serious	Not serious^q^	Not serious	Very serious^t^	Undetected^b^	No	No	No	95	203	n.s.	⨁〇〇〇 Very low
*Microfracture versus other treatments (OATS, radiofrequency ablation, and debridement) at short term (follow-up: range 6 months to 3.1 years)	318 (3 non-randomized studies)	Serious^u^	Not serious	Serious^v^	Not serious	Undetected^b^	Large^w^	Would reduce demonstrated effect^x^	No	147	171	X	⨁〇〇〇 Very low
*Microfracture versus other treatments (radiofrequency ablation and debridement) at medium term (follow-up: range 3.1 years to 8 years)	215 (2 non-randomized studies)	Not serious	Serious^y^	Not serious	Serious^z^	Undetected^b^	No	Would reduce demonstrated effect^x^	No	120	95	X	⨁〇〇〇 Very low

CI = confidence interval.

a High heterogeneity across studies (I^2^=65%, *P*=0.02). Point estimates of all studies indicate a negative effect; however, the upper limit of 95% CI of 4/5 studies is very close to 0 (≥ to −0.05), with 95% CI of one study among them containing 0.

b Impossible to evaluate publication bias due to a low number of studies (<10) compromising the body of evidence for each outcome. More information about publication bias is present in “Limitations” part of the “Discussion” section.

c Magnitude of effect expressed in SMD (Cohen’s d) value is in range of −0.30 to −0.50 units, which we classified as the medium size effect.

d A slight increase in the negative magnitude of the effect would be present if adjusted versus unadjusted values were used, based on one high-weight study. Additionally, in most studies (80% weight), the mean difference between groups before standardization was between 5% and 10%, which is moderate.

e Almost 30% of the overall weight used to calculate pooled effect estimate is contributed by Gaweda *et al*. study, graded as high bias in the adequacy of follow-up and overall domains. However, the same MA performed without biased study does not yield very different effect estimate, heterogeneity nor does it make the result statistically significant.

f Very high heterogeneity across studies (I^2^=73%, *P*=0.03). All point estimates indicate a negative effect; however, the upper limit of 95% CI of 2/3 studies (70% weight) is very close to 0 (≥–0.05).

g Optimum information size (OIS) calculation (assuming an alpha of 0.05 and a beta of 0.20) indicates a satisfactory number of participants to derive reliable conclusions. However, the 95% CI of the pooled effect estimate contains a value of 0 and is not statistically significant.

h Almost 30% of overall weight used to calculate pooled effect estimate is contributed by Gaweda *et al*. study, graded as high bias in the adequacy of follow-up and overall domains. The same MA performed without a biased study yields less negative effect estimate (–0.45 vs. –0.35 SMD) and lower heterogeneity (I^2^ 54% vs. 45%) and makes the result statistically insignificant. However, reformed MA is based on only 2 studies.

i High heterogeneity across studies (Isq=54%, *P*=0.11). Point estimates of all studies indicate a negative effect, with 2/3 studies (80%+ weight) having an upper limit of 95% CI far from the value of 0 (≤ –0.14), with 95% CI of the third study (<20% weight) not containing 0.

j High heterogeneity across studies (I^2^=55%, *P*=0.11). All point estimates indicate a negative effect, with an upper limit of 95% CI of 3/3 studies very close to 0 (≥ –0.05), with 95% CI of one study among them containing 0.

k OIS calculation (assuming an alpha of 0.05 and a beta of 0.20) a indicates satisfactory number of participants to derive reliable conclusions. However, the upper border of 95% CI of the pooled effect estimate is 0, and the *p* value of the overall effect is = 0.05.

l Three studies graded as high biased, and one as low biased overall.

m Two studies found no statistically significant correlation between the presence of concomitant cartilage lesion and OA development in ACL-reconstructed patients at short to midterm. However, one study found a negative correlation and another positive correlation between the two, both being statistically significant.

ø One study with neutral results compares OA rates in ACL-reconstructed patients with concomitant cartilage lesion to OA rates indirectly—in contralateral, uninjured knees.

n OIS calculation (assuming an alpha of 0.05 and a beta of 0.20) indicates an unsatisfactory number of participants to derive reliable conclusions. Results did not reach statistical significance in 2/4 studies. No information about the significance of the pooled effect estimate.

o One out of 3 studies included was graded as highly biased, while the other two were performed over the same cohort from NRLK, effectively counted as a single study.

p Impossible to correctly identify both the number of patients and the statistical significance of results.

q Not applicable—Heterogeneity is not accessed due to the pooled number of studies being equal to 1.

r Study results are statistically significant, with 95% CI not containing zero. However, OIS calculation (assuming an alpha of 0.05 and a beta of 0.20) indicates an unsatisfactory number of participants to derive reliable conclusions.

s In majority of studies, the mean difference between microfracture and comparator groups is 5%-10% on the respective scale used to measure outcome AND adjusted analysis is statistically significant.

t Pooled results of all studies are not statistically significant, with 95% CI of pooled effect containing zero.

u Two out of 3 studies (151/215 patients) rated as high bias, with the remaining one rated as low bias.

v In one study, microfracture is indirectly compared with the debridement procedure through no treatment group.

w In 1 of 3 studies, the mean difference between microfracture and comparator groups is between 5% and 10%, while in the remaining 2 studies, it is less than 5% on the respective scale used to measure outcome. However, a study with a moderate effect size contributes to the highest number of patients (>50%) and its adjusted analysis is statistically significant.

x In 1 study, microfracture group patients suffer from high-grade lesions (Gr 3 or 4) while radiofrequency ablation patients suffer from partial thickness lesions (Grade 1 or 2).

y One study reporting a nonsignificant difference between MF and debridement at midterm, while another reports significantly worse results of MF versus radiofrequency ablation.

z One study reports a statistically significant difference between microfracture and radiofrequency ablation group, and other insignificant difference between microfracture and debridement.

Our conclusions are supported by the SR performed by Pedersen *et al*.,^23^ which reported a correlation between cartilage lesions and worse PROMS at 2-10 years follow-up after ACL reconstruction in 31,556 subjects across 4 studies. Hamrin Senorski *et al*.^24^ also concluded that the absence of a cartilage lesion at the time of ACL reconstruction significantly increased the odds of reporting an 80th percentile result in the KOOS 4 score at 2-year follow-up. Cartilage lesion’s thickness was associated with worse KOOS scores, suggesting a relationship between the lesion thickness and PROMs. However, the similarity to Pedersen *et al*. is not surprising, considering the substantial overlap of study cohorts in both reviews.

Results were mixed in Magnussen *et al*.^22^ While Hanypsiak *et al*.^18^ and Lebel *et al*.^19^ reported no significant differences in subjective IKDC scores, Shelbourne and Gray^60^ identified articular cartilage lesion as a predictor of lower Cincinnati and subjective IKDC scores in ACL-reconstructed patients. However, the minimum follow-up time for all studies in Magnussen *et al*. was 10 years, and one of them^18^ involved patients with bone bruising at baseline.

In Fillardo *et al*.,^21^ of 27 included studies, 21 indicated worse short- to midterm subjective, objective, or radiological outcomes if a cartilage lesion was present.

Conflicting results were reported in 2 NLKR cohort studies^40,50^: where the smaller study^50^ *(n*=74) found no association between full-thickness cartilage lesions and KOOS subscale scores at 5-9 year follow-up, in contrast to the larger study^40^ (*n*=6,785). This may result from the inter-study patient characteristics, with the first^50^ using stricter inclusion criteria than the second.^40^ A younger population with higher baseline PROMs implies a more physically active cohort, plausibly with higher self-motivation and increased rehabilitation adherence.^61-63^ Another reason might be shorter injury to surgery interval between cohorts.^64,65^

Large prospective cohort studies by Rotterud^39^ and Ulstein^40^ received high relative weight in the meta-analysis models. However, considering their methodological rigor, sample size, and low bias risk, we concluded that omitting those studies would be ill-advised.

For two studies^39,40^ included in the meta-analysis, pooled crude mean KOOS QoL scores were used instead of adjusted beta coefficients, due to the intervention group structure. Adjusted and unadjusted beta coefficients were similar for partial thickness and indicated the same conclusions in the full-thickness group, which was considered during the GRADE evaluation. Consequently, we conclude that representative results are reported here.

The main limitation of the current meta-analysis is the heterogeneity in PROMs pooled across the included studies, due to limitations of the evidence base.^66^ However, all included PROMS examined similar general components of knee function, with the only exception being KOOS. Therefore, only KOOS Qol subscale scores were considered as best fit for analysis.^67,68^ Consequently, the presented analyses reflect knee function in a broader sense across multiple evaluation systems, making it difficult to give specific practical recommendations to patients with different activity levels and knee function demands.^68^

Meta-analysis comparing PROMs between patients with partial and full-thickness lesions was not performed, due to limited available evidence. However, due to frequent issues with misclassification, it is questionable whether studies focused solely on ACL-reconstructed patients with partial thickness lesions should be encouraged at all.^69-73^

A high number of studies did not report concomitant cartilage treatments,^39,42,46,47,59^ suggesting the interpretation that most lesions were untreated.

### Secondary Outcomes

We observed, with a very high degree of uncertainty, an association between cartilage lesions and both OA development and reoperation rates, in the cumulative short- and midterm (Table 8).

Conflicting results across studies may stem from the inclusion criteria, especially in regard to baseline meniscal status as a known OA predictor in the long term.^74,75^ All 3 studies^38,45,50^ that did not observe a correlation between cartilage lesions and OA development also excluded patients with meniscal lesions, unlike one study^49^ that reached the opposite conclusion. In Grassi *et al*.,^76^ both cartilage lesions and meniscal lesions were identified as independent predictors of OA development in ACL-reconstructed patients. The minimum follow-up was 20 years: since the time from surgery is critical in the pathophysiological process of OA development, we consider their study design to be more appropriate than short-term evaluations.

#### Optimal surgical cartilage lesion treatment

From the available short-term data, with high uncertainty, we observed an association between the microfracture procedure and worse PROMs compared with no treatment, and no association at midterm, with the limitation that both results used the same cohort of patients. The microfracture procedure was also associated with worse PROMs compared with OATS at short term, and radiofrequency ablation at midterm (Table 8).

One study^58^ compared PROMS in 25 ACL-reconstructed patients with full-thickness cartilage lesions treated with microfracture to 25 ACL-reconstructed patients with partial thickness cartilage lesions treated with radiofrequency ablation, attributing the differences solely to the surgical procedure, without adjusting for lesion depth as a confounder.

Another limitation was the lack of level 1 or 2 studies examining outcomes in ACL- reconstructed patients treated with novel cartilage reparation procedures (matrix-induced autologous chondrocyte implantation [MACI], scaffold- based procedures, etc.).^77^

The exact number of lesions is potentially underestimated due to: reporting only the single, highest grade lesion per patient,^38,39,48,55,59^ grouping lesions together into compartmental chondrosis,^42,43,46,47^ and unclear terminology concerning the articular cartilage injury (chondromalacia, chondrosis and focal lesions used interchangeably).^43,45-47,49,51^

Due to the small number of available studies, publication bias was not evaluated with funnel plots: some factors present suggest an increased risk of publication bias^78^ (only 1 randomized control trial [RCT], mostly observational cohort studies) or a decreased risk (large studies with rigorous methodology, no evidence of premature stopping, not a new research topic, no industry-sponsored studies).

Most studies could have described the covariate adjustment in regression analyses more precisely. There is no reasoning presented as to why certain factors are considered confounders, with no distinction between confounders, colliders, and mediators, which would be necessary for precise causal interpretations. Directed acyclic graphs (DAGs) were not created, increasing the risk of bias and preventing repeated analysis in further studies.^79-82^

The results from our study might facilitate realistic expectations following ACL reconstruction in patients with concomitant cartilage lesions, both for patients and surgeons. Finally, based on the limited body of evidence, avoiding microfracture procedures for the treatment of cartilage lesions might be advisory, considering no proven benefit compared with other, less invasive procedures.

## Conclusions

In conclusion, high-grade cartilage lesions in ACL reconstruction result in predictably worse PROMS in the short to midterm, while the same might be true regardless of the grading of the cartilage lesions. It is uncertain whether the presence of a cartilage lesion in ACL-reconstructed patients leads to knee joint OA within the relatively short time to follow-up in this SR (up to 8 years), with factors such as meniscal status potentially being decisive. Due to the scarcity of high-quality evidence, further pragmatic (RCT) studies are necessary to enable conclusions about the optimal treatment of cartilage lesions in ACL-reconstructed patients.
